# Fabrication and *in vitro* evaluation of an articular cartilage extracellular matrix-hydroxyapatite bilayered scaffold with low permeability for interface tissue engineering

**DOI:** 10.1186/1475-925X-13-80

**Published:** 2014-06-20

**Authors:** Yongcheng Wang, Haoye Meng, Xueling Yuan, Jiang Peng, Quanyi Guo, Shibi Lu, Aiyuan Wang

**Affiliations:** 1 Institute of Orthopaedics, Chinese PLA General Hospital, 28 Fuxing Road, Beijing, China; 2 Medical school of Chinese PLA, 28 Fuxing Road, Beijing, China

**Keywords:** Extracellular matrix, Hydroxyapatite, Osteochondral interface, Bilayered scaffold, Permeability

## Abstract

**Background:**

Osteochondral interface regeneration is challenging for functional and integrated cartilage repair. Various layered scaffolds have been used to reconstruct the complex interface, yet the influence of the permeability of the layered structure on cartilage defect healing remains largely unknown.

**Methods:**

We designed and fabricated a novel bilayered scaffold using articular cartilage extracellular matrix (ACECM) and hydroxyapatite (HAp), involving a porous, oriented upper layer and a dense, mineralised lower layer. By optimising the HAp/ACECM ratio, differing pore sizes and porosities were obtained simultaneously in the two layers. To evaluate the effects of permeability on cell behaviour, rabbit chondrocytes were seeded.

**Results:**

Morphological observations demonstrated that a gradual interfacial region was formed with pore sizes varying from 128.2 ± 20.3 to 21.2 ± 3.1 μm. The permeability of the bilayered scaffold decreased with increasing compressive strain and HAp content. Mechanical tests indicated that the interface was stable to bearing compressive and shear loads. Accordingly, the optimum HAp/ACECM ratio (7 w/v%) in the layer to mimic native calcified cartilage was found. Chondrocytes could not penetrate the interface and resided only in the upper layer, where they showed high cellularity and abundant matrix deposition.

**Conclusions:**

Our findings suggest that a bilayered scaffold with low permeability, rather than complete isolation, represents a promising candidate for osteochondral interface tissue engineering.

## Introduction

Articular cartilage, a stable load-bearing unit, exhibits very poor regeneration capacity if damaged due to its avascular nature and need for a precise physical environment. Current clinical methods to repair defective cartilage include microfracture, mosaicplasty, and autologous chondrocyte implantation, all of which are inadequate in their ability to regenerate functional cartilage, both in terms of composition and mechanics [1,2]. In recent years, tissue engineering has become a promising strategy to repair cartilage and osteochondral defects [3,4].

In fact, articular cartilage and the subchondral bone constitute a complex tissue structure that involves a progressive gradient of material and physiological properties [5]. The native osteochondral interface consists of a calcified cartilage layer [6] that is flanked by an undulating tidemark and an even more irregular cement line adjacent to the subchondral bone [7]. The mineral component in the calcified cartilage layer is similar to, but distinct from, that found in bone [8]. Calcified cartilage becomes established at the edges of a “permanent” epiphyseal bone layer (i.e., proximal reserve zone and articular cartilage hypertrophic zone), and the tidemark serves as a barrier to vascular invasion and the calcification of hyaline cartilage [9]. This structure is semi-permeable and permits the passage of small molecules (<500 Da) from the subchondral bone to the articular cartilage layer [10]. Moreover, this special barrier is essential for maintaining the integrity of repaired cartilage over time and preventing osseous upgrowth into full-thickness defects [11]. Thus, osteochondral interface regeneration needs more attention in repair cartilage defects through tissue engineering techniques. A number of studies have developed strategies to facilitate tissue integration at the osteochondral interface and have re-established damaged articular cartilage [12-14].

In tissue engineering, scaffolds can provide an artificial and sometimes temporary extracellular matrix (ECM), mimicking the structure and functionality of the native ECM, to physically guide or chemically inform cell responses and thus promote tissue growth [15]. Based on the native interface structure mentioned above, the ideal cartilage-to-bone interface scaffold would support chondrocyte viability and promote calcified cartilage matrix formation with appropriate physical properties. Thus, designing and fabricating such a scaffold involving a gradient interfacial structure is a prerequisite for the success of osteochondral tissue engineering.

In a previous study [16], we developed a novel articular cartilage ECM (ACECM)-derived scaffold using decellularised human joint cartilage. This scaffold could support cell attachment, proliferation, and mesenchymal stem cell differentiation and was used *in vivo* for cartilage tissue engineering [16,17]. Because cartilage-specific ECM components play an important role in chondrogenesis, as well as in supporting the chondrogenic phenotype [18,19], other biomimetic scaffolds with oriented structures were fabricated using temperature gradient-guided thermal-induced phase separation (TIPS) followed by freeze-drying to mimic the biochemical composition and natural structure of native articular cartilage [20,21]. Nevertheless, the gradient interface and permeability of the calcified layer were not considered in the scaffold designing and manufacturing process. Moreover, no study of interfacial low permeability in bilayered scaffolds for osteochondral interface regeneration has yet been reported.

In this study, we designed and fabricated a novel bilayered scaffold using ACECM and hydroxyapatite (HAp), which involved a porous oriented upper layer and a dense mineralised lower layer. The ingredient distribution and pore morphology were examined through the interface. To achieve low permeability, the porosity and pore size were regulated by the content ratio, and the permeability of the bilayered scaffold under different strain levels was evaluated. To verify the feasibility of this integrative scaffold for osteochondral interface tissue engineering, chondrocytes were then seeded on the bilayered scaffold for *in vitro* evaluation. Cell viability and distribution on the scaffold were also characterised.

## Materials and methods

### Preparation of ACECM-HAp suspensions

Integrative, bilayered scaffolds were fabricated from a mineralised ACECM and nanophase HAp (ACECM-HAp) suspension and a pure ACECM suspension. According to a method developed in our laboratory [16,21], suspensions of porcine ACECM were prepared. The cartilage slices were shattered and decellularised under aseptic conditions then smashed in PBS buffer containing 3.5% (w/v) phenylmethyl sulphonylfluoride (Merck, Darmstadt, Germany) and 0.1% (w/v) EDTA (Sigma, Poole, UK) for 60 min. The resulting suspension of cartilage fragments was then centrifuged (500 × *g*). The decellularised ACECM microfibrils were washed extensively with sterile PBS and then made into a 3% (w/v) suspension in PBS. The ACECM suspension was obtained and used without further processing. The ACECM-HAp suspension was produced by combining (porcine) decellularised ACECM microfibrillar and nanophase HAp, which was obtained commercially. Nano-sized HAp particles (mean 20 nm; DK Nano Technology, Beijing, China) were mixed with 3% (w/v) ACECM suspension at different weight to volume ratios and dispersed homogeneously in a glass container. The effect of the ceramic dose was investigated by comparing scaffolds with 3.5, 7, 10.5, or 14 w/v% HAp (i.e., 35, 70, 105, or 140 mg of HAp per mL of ACECM). A magnetic blender was used for thorough mixing for 6 h at 37°C in the incubator; the final mixture was then sonicated for 2 min. To remove any air introduced during the mixing process, both suspensions were degassed under a vacuum (<300 mTorr) at room temperature.

### Fabrication of bilayered ACECM-HAp scaffolds

The two suspension types were loaded separately into two syringes. Both scaffold components, a mineralised portion from the ACECM-HAp suspension and a non-mineralised portion from the ACECM suspension, from separate liquid precursors were used to fabricate a scaffold with a gradual coherent interface between the components formed simultaneously using the “liquid-phase cosynthesis” technique [22]. The degassed ACECM-HAp and ACECM suspensions were then injected sequentially into a custom polypropylene cylindrical mould (inner diameter, 10 mm; height, 10 mm). To make the contact intersurface more level, the layered suspensions were centrifuged at a low speed (150 rpm/min) then allowed to interdiffuse at room temperature for 30 min before subsequent processing. The layered, interdiffused suspensions were then solidified using the TIPS technique, followed by freeze-drying [21,23]. Briefly, a metal cylinder of the same diameter was embedded in the upper end of the mould (non-mineralised side), and the whole system was inverted then placed vertically onto a metal plate equilibrated to -196°C and frozen in liquid nitrogen. This technique allowed for orientation of the structure of the scaffold by solvent crystallisation under a unidirectional temperature gradient. The frozen samples were lyophilised in a freeze-dryer (Boyikang, Beijing, China) for 48 h under a vacuum.

After freeze-drying, the bilayered ACECM-HAp scaffolds were removed from the mould and cross-linked (ultraviolet light, 258 nm, 4 h) prior to immersion in 95% (v/v) alcohol containing 50 mM 1-ethyl-3-(3-dimethylaminopropyl) carbodiimide hydrochloride (EDAC) and 20 mM N-hydroxysucinimide (Sigma) for 24 h at 4°C. Excess EDAC was rinsed out of the scaffolds using PBS. The scaffolds were lyophilised then removed from the mould and cut into the required size cylinders using a biopsy punch and scalpel. Scaffold cylinders (4 mm in diameter, ~3 mm thick) from independently prepared batches were sterilised by ^60^Co γ-irradiation at 5 mRad. An additional movie file shows the production process flow in more detail [see Additional file 1].

### Structure characterisation and composition analysis

To determine the scaffold mean pore size, porosity, interconnectivity, and relative distribution of minerals throughout the scaffold, ACECM-HAp scaffold samples (*n* = 3) were analysed using micro computed tomography (microCT) with a 1-μm isotropic voxel resolution under a 60-kV scanning voltage (1072 X-ray Microtomographer; SkyScan, Kontich, Belgium). Visual inspection of each microCT frame captured (CTAn/CTVol Software Package, v.1.13/v.2.2; SkyScan, Aartselaar, Belgium) through the thickness of the scaffold allowed analysis of whether the HAp content remained distributed uniformly with the ACECM content because of the differential opacity of ACECM versus HAp by microCT.

The ACECM-HAp scaffolds (*n* = 3) were analysed by scanning electron microscopy (SEM) and energy-dispersive X-ray (EDX) spectroscopy to examine the local pore microstructure at the interface and to determine the mineral content and spatial distribution of minerals within the scaffold. Full-thickness cylindrical samples were cut in half in vertical sections, mounted on aluminium stubs, sputter-coated with gold, and observed by SEM (Hitachi BCPCAS-4800, Tokyo, Japan) at an accelerating voltage of 15 kV. Line scan and compositional map data were acquired via EDX spectroscopy for the calcium (Ca) and phosphorous (P) contents as well as other elements.

### Fourier transform infrared (FTIR) spectroscopy

The presence of nanoparticles and the resulting chemical composition of the ACECM-HAp scaffolds (*n* = 5) were analysed by FTIR spectroscopy (Spectrum One; Perkin Elmer, Waltham, MA, USA) in transmission mode. Transparent potassium bromide pellets were prepared, followed by uniaxially pressing the powders under a vacuum. All spectra were obtained between 4400 and 450 cm^-1^ at a 2-cm^-1^ resolution. A dry system was used to prevent atmospheric moisture.

### Unconfined and confined compressive testing

The bilayered scaffolds (*n* = 6) were placed into a confined compression chamber and attached to a standard materials testing machine with a 225-N load cell (ElectroForce 3220; BOSE, Eden Prairie, MN, USA). A preload of 0.05 N was applied to ensure contact between the scaffold surface and the porous indenter. To ensure that the tissues were fully hydrated, the samples were kept immersed in PBS until testing. Mechanical tests were performed at a constant compression rate of 0.05 mm/s to a maximum strain of 35%. The same-sized single-layer (ACECM and ACECM-HAp) scaffolds were put into the confined compression chamber and tested with the test sequence described previously. For unconfined compression testing, bilayered or single-layer scaffolds (*n* = 6) were positioned between two permeable steel platens. The same testing regime as that described for confined testing was used.

### Interface shear strength test

The interfacial shear properties of the bilayer scaffolds (*n* = 6) were assessed using a custom shear holding device held in the testing machine. Scaffolds were kept hydrated by immersion in a PBS bath at room temperature then placed in the shear apparatus and adjusted so that a metal shearing plate contacted the tissue ~0.5 mm from the bilayered scaffold interface. The tests involved a 0.02 mm/s crosshead velocity. The peak load at failure was taken as the highest point corresponding to the first observed peak on the load–displacement curve, and the shear stiffness was calculated using the slope of the steepest and most linear portion of the curve up to this peak load.

### Permeability analysis

**Figure 1:**
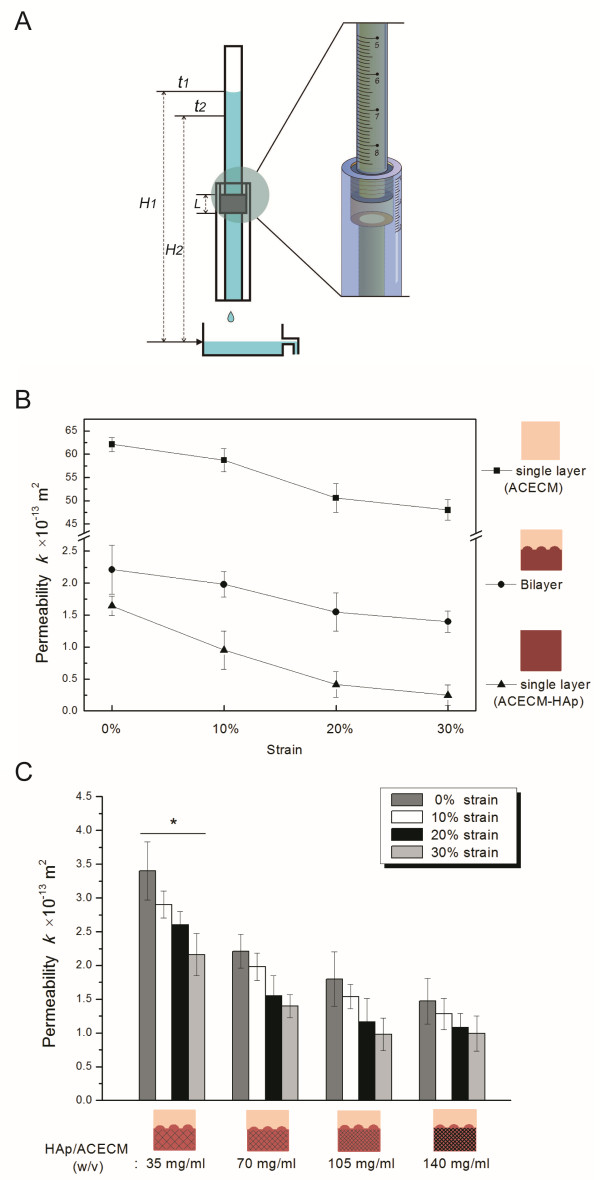
**Permeability determination. A**: Experimental set-up for the measurement of permeability. **B**: Permeability changes in different types of scaffolds (7 w/v% HAp) under compressive strain. **C**: Experimentally measured permeability of bilayered scaffolds with various HAp contents and applied compressive strain. The data are means ± SDs, * *p* < 0.05, versus respective bilayered scaffolds at the same strain.

The specimens tested for permeability included all ACECM scaffolds and ACECM-HAp scaffolds as well as the bilayered ACECM-HAp scaffolds (*n* = 5, each). Permeability was determined by a falling-head conductivity test based on Darcy’s law [24]. A custom chamber device was constructed to provide different levels of compressive strain on the scaffolds and to measure the variation in fluid height. The device consisted mainly of 1) a standpipe to provide a falling head, 2) a permeability chamber to host the scaffold and adjust the levels of applied compressive strain (with a scale in mm), and 3) a reservoir for fluid collection (Figure 1A).

Two stainless steel meshes were adhered to the openings of the standpipe and chamber to ensure uniform compressive strain on the scaffold. The mesh did not inhibit or disrupt fluid flow through the tube. The standpipe, with a 7.5-mm inner diameter, could be screwed into the permeability chamber, for a volume 8.0 mm in diameter, for the host sample. The level of compressive strain was regulated by adjusting the depth of the standpipe screw. Cylindrical samples (8.4 mm in diameter, 7.0 mm thick) were cut from the various scaffolds using a corneal punch. Saline solution (0.9% NaCl) was used as the fluid medium for the permeability test. Before measurement, the samples were submerged in saline solution for 1 h under negative pressure to remove air bubbles trapped in the structure.

As shown in Figure 1A, the initial height (*H*_1_) of fluid in the standpipe was recorded at time 1 (*t*_1_). While liquid permeated through the scaffold, the time (*t*_2_) required for the fluid head to drop from the upper to the lower level (*H*_2_) was recorded using a stopwatch. The permeability (*k*) and hydraulic conductivity (*K*) of the scaffold under each level of applied compressive strain were calculated on the basis of Darcy’s law [24]:

(1) $$k=K\frac{\mu }{\rho g}$$

and

(2) $$K=\frac{a}{A}\frac{L}{t}\ln\frac{H_{1}}{H_{2}}$$

where *μ* is the dynamic viscosity, *ρ* is the fluid medium density, *a* is the cross-sectional area of the standpipe, *A* is the cross-sectional area of the sample, and *L* is the sample thickness under compression. To obtain reproducible results, the permeability was measured five times for each sample.

### Isolation of chondrocytes and cell seeding on the scaffolds

Rabbit chondrocytes were obtained from New Zealand White rabbits as described previously [25]. The protocol was approved by the Institutional Animal Care and Use Committee of Chinese PLA General Hospital (Beijing, China). The cells were cultured and expanded in regular culture medium (DMEM supplemented with 10% foetal bovine serum, 300 mg/mL L-glutamine, 50 mg/mL vitamin C, 100 U/mL penicillin, and 100 U/mL streptomycin) and passaged three times (P3) before use.

The bilayer scaffolds (4 mm in diameter, 3 mm thick; *n* = 18) were sterilised with ethylene oxide and placed into a 6-well culture plate rinsed with medium for 20 min, and 35-μL cell suspensions of ~1 × 10^5^ cells were seeded onto each sample until the scaffold became completely saturated. The cell-scaffold constructs were then incubated for 4 h at 37°C at 95% humidity/5% CO_2_ to allow for complete adhesion of the cells to the scaffolds. Next, the constructs were covered with pre-warmed regular culture medium and cultured under the same conditions. The medium was replaced every 2–3 days and the constructs were harvested for further evaluation.

### Cell behaviour: observation of the bilayered scaffold

Cell attachment was confirmed by direct visualisation of the constructs using SEM (*n* = 3) after 7 days in culture. The cell-scaffold constructs were fixed in 2.5% glutaraldehyde, dehydrated through a graded ethanol series to 100% ethanol, treated with hexamethyldisilazane, and sputter-coated with gold/palladium before viewing. Histological examination of the cell-scaffold constructs was also performed using haematoxylin and eosin (H&E), safranin O, toluidine blue, and alizarin red staining.

Cell viability in the scaffolds was evaluated at 3, 7, and 14 days (*n* = 3 each) using a Live/Dead assay kit (fluorescein diacetate [FDA]-propidium iodide [PI]; Sigma). Live cells were stained with FDA and dead cells were labelled with a working solution of PI for 5 min. The stained constructs were viewed under an Olympus IX81 confocal microscope (Tokyo, Japan).

### Statistical analyses

All data are presented as means ± SDs. A one-way analysis of variance and *post hoc* Student-Newman-Keuls test were used to assess differences in the porosity data, biomechanical data, and permeability data. *P*-values < 0.05 were considered to indicate statistical significance.

## Results

### Structural characterisation of the bilayered scaffolds

**Figure 2:**
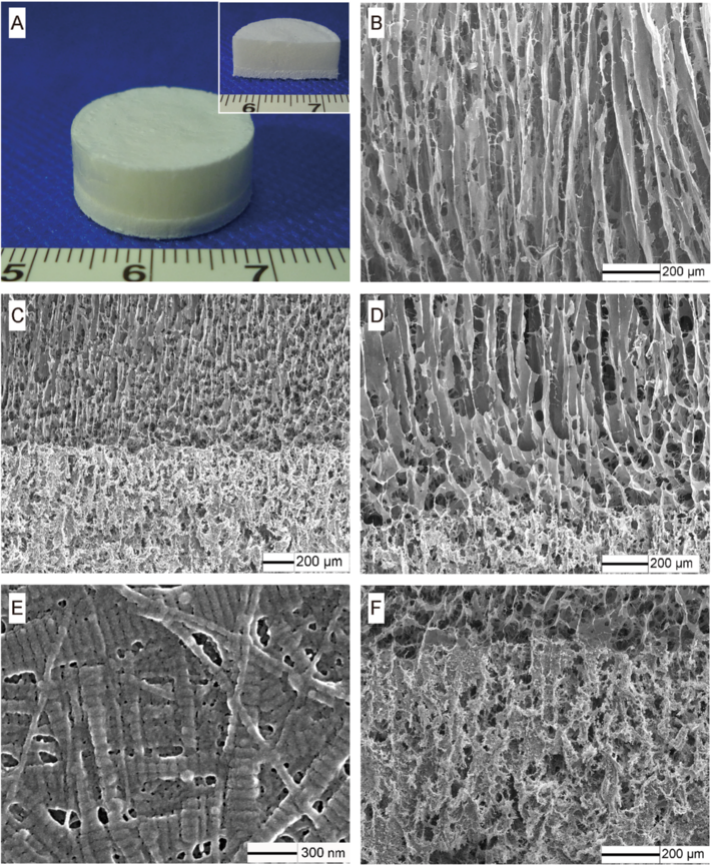
**Structural characterisation of the bilayered scaffolds. A**: Gross appearance of the scaffold prototype. **B–F**: Analysis of longitudinal sections of the bilayered scaffold by SEM showing the upper layer **(B)**, interface region **(C and D)**, and nanofibrous ECM orientation in the upper layer of the scaffold wall at a high magnification **(E)** and in the lower layer **(F)**.

The manufactured scaffolds matched the original design. Macroscopic (Figure 2A) and electron microscopic observations (Figure 2B) of the bilayered prototype revealed a remarkable area of continuity at the interface. Thus, the two layers showed good combining characteristics, a requirement necessary to ensure excellent integrity and functionality of the interfacial construct. The pore structures of the upper and lower layers were distinct. The pores within the upper layer were microtubule-like and arranged in parallel in longitudinal sections (Figure 2B–D), each with a characteristic three-dimensional (3D) interconnected pore structure. The nanofibrous ACECM aligned along the longitudinal orientation in the vertical section image at a high magnification (Figure 2E). Distinct from the upper layer, the lower layer was denser, with an arbitrary composition; nanophase HAp particles were uniformly distributed throughout the local region and showed a lack of crannies, regions of delamination, or any other defects at the interface (Figure 2F).

**Figure 3:**
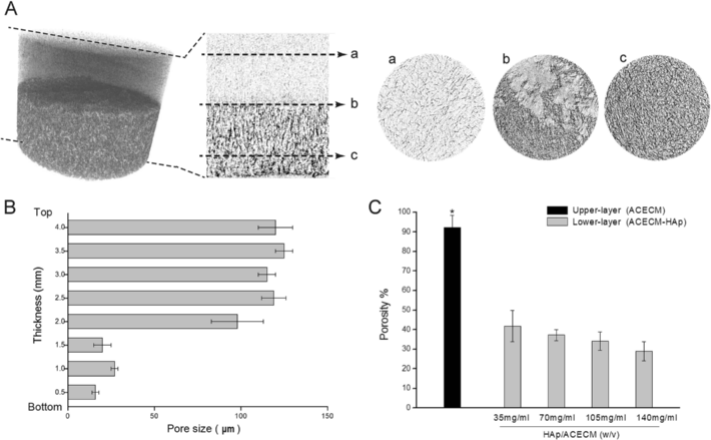
**Architecture characterisation. A**: Analysis of cross-sectional images of the bilayered scaffold by microCT showing the upper layer (a), interface region (b), and lower layer (c) of the scaffold (position marked by a dashed line). **B**: Pore size distribution at distinct thicknesses. **C**: Porosity of the upper and lower layers with various HAp contents. The data are means ± SDs. *p < 0.05.

The scaffold structure and porosity were also assessed by microCT. Images of the bilayered scaffold microstructure are shown in Figure 3A (side profile and three transverse cross-sections through the scaffold disk taken from the upper, interfacial, and lower regions). The translucent upper layer (non-mineralised) was clearly distinguishable from the HAp-free component and the more opaque lower layer (mineralised) was the HAp-containing component. An analysis of serial cross-sections (*n* = 6 per scaffold component) of the microCT images (Figure 3B) allowed calculation of the mean pore size in the upper and lower layers. The pores in both the mineralised and non-mineralised components were multidimensional, with mean pore sizes of 128.2 ± 20.3 and 21.2 ± 3.1 μm, respectively. The difference between the mean pore sizes of the two layers revealed that different pore structures can be obtained using the current methods. The material porosity of the bilayered scaffold was calculated using SkyScan CTAn Software with microCT and indicated a porosity of 92.6 ± 6% in the non-mineralised components and from 44 ± 3 to 30 ± 4% (with 3.5, 7, 10.5, or 14 w/v% HAp) in the mineralised components (Figure 3C). Also, the upper layer showed larger porosity than the lower layers (*p* = 0.001). Likewise, imaging of the interfacial region of the bilayered scaffold by microCT showed, as did SEM (Figure 2C and D), a lack of crevices or discontinuities at the interface (Figure 3A).

### Compositional analysis and FTIR spectroscopy

**Figure 4:**
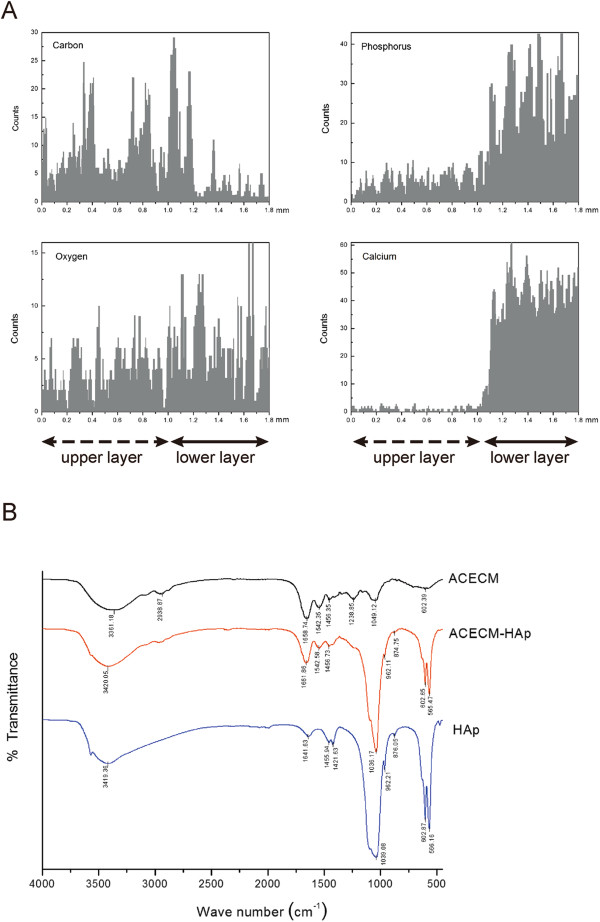
**Compositional analysis. A**: EDX analysis of the bilayered scaffold; solid and dashed arrows highlight the mineralised (upper layer) and unmineralised (lower layer) compartments, respectively. **B**: FTIR measurements of the cross-linked ACECM scaffold, ACECM-HAp (with 7 w/v% HAp) bilayered scaffold, and non-cross-linked nanophase HAp crystal particles.

Distinct mineralised (high calcium [Ca] and P contents) and non-mineralised (low to non-existent Ca and P contents) regions were revealed within the scaffold via EDX analysis of the bilayered scaffold (from the top, through the thickness), corresponding to the known locations of the ACECM-HAp and ACECM scaffold components (Figure 4A). A uniform distribution was observed within the lower layer, whereas little or no mineral content was observed in the upper layer. Quantification of the mineral (Ca or P) content through the thickness of the bilayered scaffold showed a high concentration of minerals in the lower layer that was almost two orders of magnitude greater than the mineral content in the upper layer, which was negligible (Figure 4A, right side). Furthermore, the distribution of carbon and oxygen (Figure 4A, left side) indicated no angular interface between the two layers.

FTIR spectroscopy can be an effective approach to define the existence of each component within a complex system containing different types of materials. Figure 4B shows the FTIR spectra of the non-cross-linked nanophase HAp crystal particles and cross-linked ACECM scaffold as well as the ACECM-HAp bilayered scaffold with 7 w/v% HAp. Typical infrared bands showed characteristic phosphate (PO_4_^3-^) absorption bands at 566.16, 602.87, and 1039.08 cm^-1^, indicating HAp nanoparticles, which were not observed in the ACECM-only spectrum. The characteristic absorption bands of ACECM were observed at 1658.74 (amide I), 1542.35 (amide II), and 1238.85 cm^-1^ (amide III). Nevertheless, the FTIR spectra of cross-linked ACECM-HAp showed no new adsorption band in any range; the peaks were shifted slightly with respect to the standard values for PO_4_^3-^ adsorption bands, which may be due to the interaction between the ceramic and the collagen phase [24]. This finding is probably the result of the material preparation and cross-linking methods not affecting their chemical environment.

### Mechanical properties

**Figure 5:**
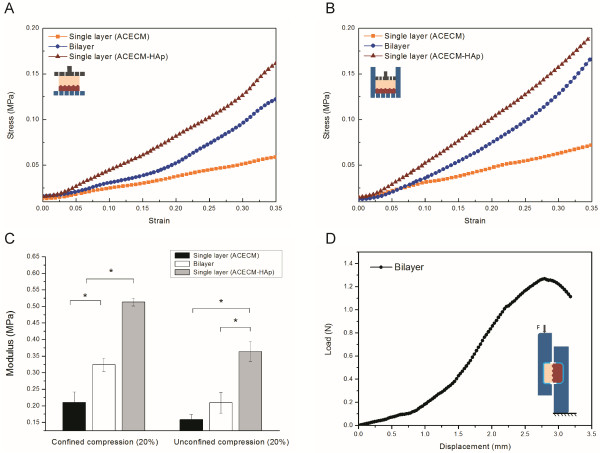
**Mechanical properties. A**: Unconfined compression of the single-layer and bilayered scaffolds. **B**: Confined compression of the single-layer and bilayered scaffolds. **C**: Compressive moduli in confined and unconfined compression (20% strain) of various scaffolds. The data are means ± SDs. *p < 0.05. **D**: Typical shear load–displacement curve of the interfacial zone of a bilayered scaffold.

Typical stress–strain curves for three different forms of the scaffolds obtained from both confined and unconfined compression tests are shown in Figure 5A and B. The compressive modulus was determined from the slope of the linear elastic stage in the curve. The single-layer (ACECM) scaffolds had a relatively low compressive modulus (Figure 5C), whereas the single-layer (ACECM-HAp) scaffolds had a significantly higher compressive modulus compared with the other groups under confined and unconfined situations. The modulus of the bilayered scaffolds ranged between the values of the two single-layer scaffolds. Thus, the addition of HAp to the ACECM construct increased the compressive modulus, and the scaffold structure significantly affected the mechanical capacity.

A representative shear load–displacement curve of the bilayered scaffold is shown in Figure 5D; corresponding values for shear stiffness and peak load at fracture on different HAp-content scaffolds are shown in Table 1. The interface shear stiffness was calculated by determining the maximum slope of the most linear portion of the load–displacement curve for each sample. The interfacial shear resistance of the bilayered scaffold decreased with increasing HAp content in the lower layer. The shear stiffness was higher in the scaffolds containing a low HAp content than in the scaffolds containing a high HAp content (*p* < 0.001).

**Table 1:** Interfacial shear properties of bilayered scaffold with various HAp content in hydrated status

	**HAp**_**35 mg/mL**_	**HAp**_**70 mg/mL**_	**HAp**_**105 mg/mL**_	**HAp**_**140 mg/mL**_
**Peak load (N)**	1.33 ± 0.21	0.86 ± 0.06	0.54 ± 0.10	0.28 ± 0.04
**Shear stiffness (N/mm)**	1.28 ± 0.13	0.79 ± 0.09	0.41 ± 0.05	0.22 ± 0.08

### Permeability

The permeability (Figure 1A) of the single-layer (ACECM) scaffold ranged from 62.1 × 10^-13^ to 48.7 × 10^-13^ m^2^ with increasing compressive strain from 0 to 30%, but in the bilayered scaffold, regardless of the concentration of HAp, the permeability was significantly smaller compared with the single-layer (ACECM) scaffold (Figure 1B). The permeability of the bilayered scaffold with 0.07 mg/mL HAp decreased from 2.2 × 10^-13^ to 1.4 × 10^-13^ m^2^ with increasing compressive strain, and that of the single-layer (ACECM-HAp) scaffold decreased from 1.6 × 10^-13^ to 0.2 × 10^-13^ m^2^ with increasing compression (Figure 1B). In contrast, the permeabilities of the bilayered scaffold and the single-layer (ACECM-HAp) scaffold without strain were similar, with no significant difference between the two scaffolds in the rate of decline under strain. With the bilayered scaffold, the permeability was affected by the HAp content (Figure 1C). Generally, the value decreased with an increasing HAp content under various compressions.

### Cell morphology on the scaffold

**Figure 6:**
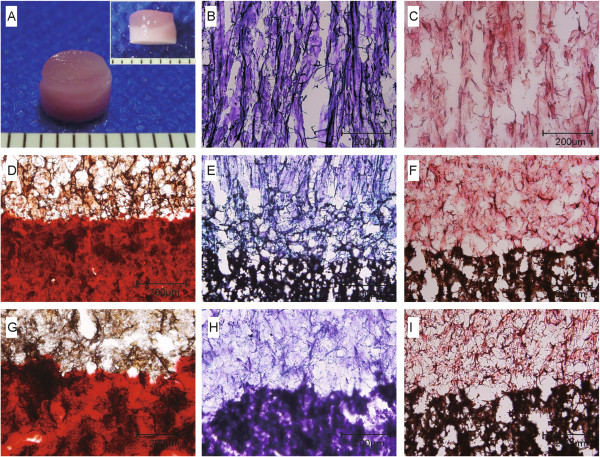
**Chondrocytes seeded on bilayered scaffolds after 7 days of culture. A**: Gross observation. **B** and **C**: Upper layer. **D–F**: Interfacial zone and lower layer. **G–I**: Interfacial zone and lower layer of unseeded scaffold as control. **D** and **G**: Alizarin red staining. **B**, **E**, and **H**: Toluidine blue staining. **C**, **F**, and **I**: Safranin O staining.

**Figure 7:**
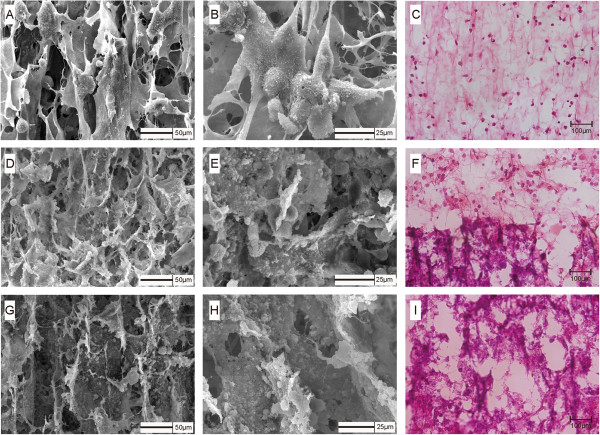
**Cell distribution of tissue engineered constructs after 7 days in culture. A–C**: Upper layer. **D–F**: Interfacial zone. **G–I**: Lower layer. **A**, **B**, **D**, **E**, **G**, and **H**: Images produced by SEM. **C**, **F**, and **I**: H&E staining.

**Figure 8:**
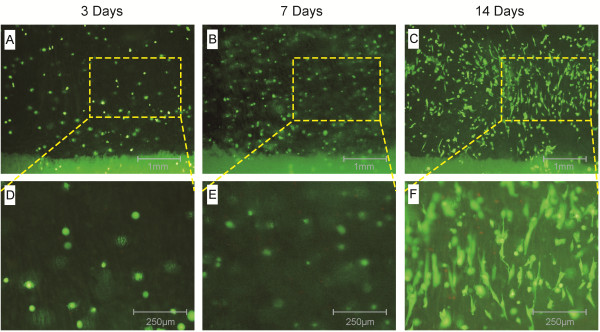
**Live/dead staining of bilayered scaffolds seeded with chondrocytes. A** and **D**: Cells cultured for 3 days. **B** and **E**: Cells cultured for 7 days. **C** and **F**: Cells cultured for 14 days. **D–F**: High magnification view of a rectangular area in **A–C**. Green: live cell staining; Red: dead cell staining.

After *in vitro* culture, a zonal, shiny cartilage-like tissue at the macroscopic level was generated when rabbit chondrocytes were seeded onto ACECM-HAp scaffolds for 7 days. The scaffold seeded with chondrocytes was cartilage-like tissue in gross view (Figure 6A). Histological staining showed all upper layers of the constructs were intensely stained with safranin O and toluidine blue, indicating an ECM rich in sulphated proteoglycans (Figure 6B and C), while the lower layers of the constructs (Figure 6E and F) indicated overexpression due to the presence of mineralised Ca and P, as in the unseeded scaffolds (Figure 6H and I). Positive alizarin red staining of the lower layers indicated the rich Ca content in both the cell-scaffold constructs (Figure 6D) and the unseeded scaffolds (Figure 6G), suggesting the mineralization of the materials, while the upper layers of the constructs were negative for staining due to the absence of Ca and P. SEM and H&E staining (Figure 7) showed that the chondrocytes were well-distributed in the non-mineralised component (Figure 7A–C) of the bilayered scaffolds, with a few cells adhering to the interfacial zone (Figure 7D–F), although no cells entered into the mineralised component (Figure 7G–I), indicating a cell-barrier layer. Figure 8 shows the cell viability of rabbit chondrocytes seeded on the bilayered scaffolds with FDA and PI staining after culture for 3, 7, and 14 days. On day 3, the upper layer exhibited an even cellular distribution with many live cells (Figure 8A and D). With increasing incubation time, more live cells could be observed aligned along the microtubules on day 7 (Figure 8B and E). Up to 2 weeks, more cells showed a fusiform morphology, were in good condition, and were aligned along vertical microtubules and adapted to upper layers of the scaffold (Figure 8C and F). However, dead cells were evident too; a few adhered to the scaffolds. The cell distribution was consistent with our findings by SEM, especially the finding that few cells infiltrated the lower layer.

## Discussion

Interfacial tissue engineering is a promising strategy for osteochondral defect regeneration, but it is challenging to fabricate a suitable tissue engineered scaffold because of the complex interfacial structure and varying composition. In this study, we used ACECM combined with HAp to fabricate a novel bilayered scaffold involving a porous oriented upper layer and a dense mineralised lower layer. In fact, the performance of the pore architecture can significantly affect cellular responses and *in vivo* bioactivity [22]. This bilayered scaffold showed a uniform pore microstructure and high interconnectivity. The pores in the upper layer were microtubule-like and arranged in parallel in vertical sections (Figure 2A–C). Notably, the pore size between the two layers decreased gradually, and the pore morphology changed to a round shape, indicating an apparent transition region, similar to a natural calcified cartilage layer [9].

We reported previously that ACECM scaffolds exhibited good biocompatibility for supporting cell attachment and proliferation in *in vitro* and *in vivo* studies [16,20]. Additionally, recent findings indicated that the proportion of HAp as an inorganic compound in the calcified cartilage layer was ranked only second to that of subchondral bone [8]. In the present bilayered scaffold, the mineralised layer consisted of a mixture of ACECM and HAp while the non-mineralised layer consisted of ACECM alone. Using low-speed centrifugation in the preparation process can distribute the HAp in the ACECM with a gradient concentration, as confirmed by our EDX spectroscopic results (Figure 4).

Viewed from the natural cartilage structure, the calcified layer plays an important role in the transmission of substance and signals, leading to different niches on both sides, for chondrocyte and osteoblast growth. Some studies have shown that bone marrow-derived mesenchymal stem cells (MSCs), which are recruited from the marrow cavity to a cartilage defect site during the healing process, resulted in poor long-term effects [26,27]. Lories *et al.*[28] also demonstrated that the calcified layer served as a vital physical barrier between the cartilage and subchondral bone, providing the articular cartilage with an independent environment. We conclude that low permeability, rather than complete isolation, at the interface is beneficial for cartilage reconstruction. Cell migration from both sides should be prevented, but nutritional and other factors can be straight for wardly transported between the layers. Accordingly, this bilayered structure was designed to provide ideal niches for different functional layer formation. The mean pore size of the lower layer in this structure was controlled within 20 μm (Figure 3B), which could prevent cell movement. Transport within the scaffold was assessed by measurement of the permeability, and the results corresponded largely with our expectations. The permeability of the bilayered scaffold differed significantly from that of the single-layer (ACECM) scaffold. However, the differences in construct permeability suggested significantly higher levels of metabolite diffusion in the upper layer, allowing increased cell proliferation and facilitating cell migration to the interface. An *in vitro* study indicated that greater scaffold permeability enhanced chondrogenic differentiation of bone marrow MSCs, whereas less permeable scaffolds were favourable for chondrocyte growth [29].

Permeability is an effective parameter in evaluating tissue engineering scaffolds that could reflect important parameters such as the combination of porosity, pore size and distribution, interconnectivity, fenestration size and distribution, and pore orientation [30]. Several experimental methods have been developed to test scaffold permeability according Darcy’s law and/or the Forchheimer equation [31]. The falling head conductivity test is a gravity-based direct method. Lee *et al*. [32] evaluated the permeability of 3D porous poly (propylene fumarate) scaffolds with a controlled pore size architecture using the falling head method. Additionally, Al-Munajjed *et al*. [33] analysed how pore size influenced the permeability of hyaluronic-collagen scaffolds. Mansour and Mow [34] found that the permeability of cartilage ranged from 0.1 to 2 × 10^-15^ m^2^ while cartilage permeability depended on the composition, tissue depth, and mechanical conditions. Cartilage permeability decreased with increasing tissue depth, as far as the calcified layer. For cartilage regeneration, the effect of compressive strain on scaffold permeability is an important feature to determine because many tissue engineering constructs are used in loading-bearing applications that can significantly influence construct permeability, as well as metabolite diffusion, but few studies have used this method to evaluate permeability. The scaffold permeability data under compressive strain in this work show that the permeability decreased with increasing strain, similar to the relationship between permeability and compression found by Lai and Mow for articular cartilage [35]. O’Brien *et al*. [36] found that collagen scaffold permeability increased with increasing pore size and decreased with increasing compressive strain, which was also supported by a mathematical analysis.

The native calcified cartilage region forms an interface with the underlying subchondral bone, and this transitional region may generate substantial shear stress because of the large discrepancy in tissue stiffness [37]. As the mechanical test results showed, determination of the compressive modulus of the bilayered scaffold demonstrated that the presence of this mineralised zone improved the compressive performance versus a scaffold without a mineralised zone. This finding was attributed to the more submissive non-mineralised layer undergoing large deformations under compressive loading, which resulted in almost total densification of the upper zone at stresses insufficient to cause observable deformation in the lower zone. Additionally, the shear strength of the interface was determined and the results were competent compared with other reports [38,39]. In all specimens, the low HAp content groups (35 and 70 mg/mL) performed better than the high HAp content groups (105 and 140 mg/mL), in which a fracture occurred in the lower layer. With regard to permeability, 70 mg/mL HAp was the most suitable candidate for calcified cartilage formation. Additionally, this bilayered structure could provide good initial stability and mechanical support during implantation. Our aim in the present study was to develop a novel scaffold that would have distinct yet continuous matrix regions. Successful realisation of this goal would be a significant advance towards developing interface tissue engineering solutions for soft-hard tissue integration and biological fixation.

The promising utility of bilayered scaffold for tissue engineering depends on the capacity to direct cells to express a desired phenotype in specific niches. Additionally, a prerequisite for biomimetic scaffold design is a clear understanding of the structure-function relationship at the cartilage-to-bone interface. The cell affinity of the scaffold is important for tissue regeneration and functional reconstruction [40]. We found that cell proliferation was maintained throughout the culture period. SEM revealed that the chondrocytes aligned vertically along the microtubules in the upper layer, but that few cells adhered to the interfacial zones, and no cells entered into the lower layer (Figure 7). Due to restrictions in the structure of the scaffold, cells cannot penetrate the mineralised layer, and cultured chondrocytes may not affect the deposition of Ca and P. Based on previous studies [41-43], as an osteoconductive material, HAp ceramics have the ability to provide an appropriate scaffold or template for bone formation; it has been used extensively as a substitute in bone grafts because the crystalline phase of natural bone is basically HAp. From the point of view of these applications in combination with our results, we hypothesise that the mineralised region of the bilayered scaffold may promote bone formation via an *in situ* tissue regeneration process [44,45]. For tissue engineering applications, a controlled scaffold orientation is needed because the alignment of the scaffold guides cellular growth and spatial alignment [46]. In this study, the cell distribution was biomimetic to the native physiological structure of deep zone cartilage and the calcified zone.

## Conclusions

In summary, we designed and fabricated an ACECM-HAp bilayered scaffold containing a porous, oriented upper layer and a dense, mineralised lower layer. A gradual interfacial region was formed by optimising the HAp/ACECM ratio. Different porosities and pore sizes of the two layers resulted in low permeability, rather than complete isolation, which could promote the formation of a local microenvironment and avoid delamination. The mechanical properties and observed cell affinity demonstrate that this bilayered scaffold could represent a promising candidate for cartilage interface tissue engineering applications. To better understand the function of this bilayered scaffold for interface reconstruction, a comparative evaluation of the different cell activities in both layers is needed. Future studies should focus on using representative animal models to explore the feasibility of *in vivo* applications. The key factors of the culture conditions (i.e., dynamic bioreactor cultivation) are also worthy of investigation.

## Abbreviations

ACI: Autologous chondrocyte implantation; ECM: Extracellular matrix; ACECM: Articular cartilage extracellular matrix; TIPS: Thermal induced phase separation; HAp: Hydroxyapatite; SEM: Scanning electron microscopy; EDX: Energy dispersive X-ray; FTIR: Fourier transform infrared; MSCs: Mesenchymal stem cells.

## Competing interests

The authors declare that they have no competing interests.

## Authors’ contributions

YW: composed the manuscript, HM and XY: analyzed the data and worked on the methods, SL and AW: proposed the idea, YW, JP and QG: made the discussions. All authors read and approved the final manuscript.

## Supplementary Material

Additional file 1 Flow chart for preparation of suspensions and fabrication of bilayered scaffolds. Click here for file

## References

[B1] Bedi A Feeley BT Williams RJ 3rd Management of articular cartilage defects of the knee J Bone Joint Surg Am 2010 92 994 1009 20360528 10.2106/JBJS.I.00895

[B2] Cole BJ Pascual-Garrido C Grumet RC Surgical management of articular cartilage defects in the knee Instr Course Lect 2010 59 181 204 20415379

[B3] Johnstone B Alini M Cucchiarini M Dodge GR Eglin D Guilak F Madry H Mata A Mauck RL Semino CE Stoddart MJ Tissue engineering for articular cartilage repair–the state of the art Eur Cell Mater 2013 25 248 267 23636950 10.22203/ecm.v025a18

[B4] Nukavarapu SP Dorcemus DL Osteochondral tissue engineering: current strategies and challenges Biotechnol Adv 2013 31 706 721 23174560 10.1016/j.biotechadv.2012.11.004

[B5] Mohan N Dormer NH Caldwell KL Key VH Berkland CJ Detamore MS Continuous gradients of material composition and growth factors for effective regeneration of the osteochondral interface Tissue Eng A 2011 17 2845 2855 10.1089/ten.tea.2011.0135 21815822

[B6] Yang PJ Temenoff JS Engineering orthopedic tissue interfaces Tissue Eng B Rev 2009 15 127 141 10.1089/ten.teb.2008.0371 PMC2817661 19231983

[B7] Wang F Ying Z Duan X Tan H Yang B Guo L Chen G Dai G Ma Z Yang L Histomorphometric analysis of adult articular calcified cartilage zone J Struct Biol 2009 168 359 365 19723582 10.1016/j.jsb.2009.08.010

[B8] Zhang Y Wang F Tan H Chen G Guo L Yang L Analysis of the mineral composition of the human calcified cartilage zone Int J Med Sci 2012 9 353 360 22811609 10.7150/ijms.4276 PMC3399215

[B9] Hoemann CD Lafantaisie-Favreau CH Lascau-Coman V Chen G Guzman-Morales J The cartilage-bone interface J Knee Surg 2012 25 85 97 22928426 10.1055/s-0032-1319782

[B10] Arkill KP Winlove CP Solute transport in the deep and calcified zones of articular cartilage Osteoarthritis Cartilage 2008 16 708 714 18023368 10.1016/j.joca.2007.10.001

[B11] Hunziker EB Driesang IM Saager C Structural barrier principle for growth factor-based articular cartilage repair Clin Orthop Relat Res 2001 391 Suppl S182 S189 11603703 10.1097/00003086-200110001-00018

[B12] Ding C Qiao Z Jiang W Li H Wei J Zhou G Dai K Regeneration of a goat femoral head using a tissue-specific, biphasic scaffold fabricated with CAD/CAM technology Biomaterials 2013 34 6706 6716 23773816 10.1016/j.biomaterials.2013.05.038

[B13] Khanarian NT Haney NM Burga RA Lu HH A functional agarose-hydroxyapatite scaffold for osteochondral interface regeneration Biomaterials 2012 33 5247 5258 22531222 10.1016/j.biomaterials.2012.03.076 PMC3786874

[B14] Dormer NH Busaidy K Berkland CJ Detamore MS Osteochondral interface regeneration of rabbit mandibular condyle with bioactive signal gradients J Oral Maxillofac Surg 2011 69 e50 e57 21470747 10.1016/j.joms.2010.12.049 PMC3101307

[B15] O’Shea TM Miao X Bilayered scaffolds for osteochondral tissue engineering Tissue Eng B Rev 2008 14 447 464 10.1089/ten.teb.2008.0327 18844605

[B16] Yang Q Peng J Guo Q Huang J Zhang L Yao J Yang F Wang S Xu W Wang A Lu S A cartilage ECM-derived 3-D porous acellular matrix scaffold for in vivo cartilage tissue engineering with PKH26-labeled chondrogenic bone marrow-derived mesenchymal stem cells Biomaterials 2008 29 2378 2387 18313139 10.1016/j.biomaterials.2008.01.037

[B17] Kang H Peng J Lu S Liu S Zhang L Huang J Sui X Zhao B Wang A Xu W Luo Z Guo Q In vivo cartilage repair using adipose-derived stem cell-loaded decellularized cartilage ECM scaffolds J Tissue Eng Regen Med 2014 8 442 453 22674864 10.1002/term.1538

[B18] Martel-Pelletier J Boileau C Pelletier JP Roughley PJ Cartilage in normal and osteoarthritis conditions Best Pract Res Clin Rheumatol 2008 22 351 384 18455690 10.1016/j.berh.2008.02.001

[B19] Benders KE Weeren PR Badylak SF Saris DB Dhert WJ Malda J Extracellular matrix scaffolds for cartilage and bone regeneration Trends Biotechnol 2013 31 169 176 23298610 10.1016/j.tibtech.2012.12.004

[B20] Zheng XF Lu SB Zhang WG Liu SY Huang JX Guo QY Mesenchymal stem cells on a decellularized cartilage matrix for cartilage tissue engineering Biotechnol Bioproc E 2011 16 593 602

[B21] Zheng X Yang F Wang S Lu S Zhang W Liu S Huang J Wang A Yin B Ma N Zhang L Xu W Guo Q Fabrication and cell affinity of biomimetic structured PLGA/articular cartilage ECM composite scaffold J Mater Sci Mater Med 2011 22 693 704 21287238 10.1007/s10856-011-4248-0

[B22] Harley BA Lynn AK Wissner-Gross Z Bonfield W Yannas IV Gibson LJ Design of a multiphase osteochondral scaffold III: Fabrication of layered scaffolds with continuous interfaces J Biomed Mater Res A 2010 92 1078 1093 19301263 10.1002/jbm.a.32387

[B23] Yang F Qu X Cui W Bei J Yu F Lu S Wang S Manufacturing and morphology structure of polylactide-type microtubules orientation-structured scaffolds Biomaterials 2006 27 4923 4933 16759695 10.1016/j.biomaterials.2006.05.028

[B24] Levick JR Flow through interstitium and other fibrous matrices Q J Exp Physiol 1987 72 409 437 3321140 10.1113/expphysiol.1987.sp003085

[B25] Yoon YM Kim SJ Oh CD Ju JW Song WK Yoo YJ Huh TL Chun JS Maintenance of differentiated phenotype of articular chondrocytes by protein kinase C and extracellular signal-regulated protein kinase J Biol Chem 2002 277 8412 8420 11744731 10.1074/jbc.M110608200

[B26] Csaki C Schneider PR Shakibaei M Mesenchymal stem cells as a potential pool for cartilage tissue engineering Ann Anat 2008 190 395 412 18842397 10.1016/j.aanat.2008.07.007

[B27] Gelse K Endochondral ossification in cartilage repair tissue hampers bone marrow stimulating techniques Rheumatol Curr Res 2012 S3 2

[B28] Lories RJ Luyten FP The bone-cartilage unit in osteoarthritis Nat Rev Rheumatol 2011 7 43 49 21135881 10.1038/nrrheum.2010.197

[B29] Kemppainen JM Hollister SJ Differential effects of designed scaffold permeability on chondrogenesis by chondrocytes and bone marrow stromal cells Biomaterials 2010 31 279 287 19818489 10.1016/j.biomaterials.2009.09.041

[B30] Li S De Wijn JR Li J Layrolle P De Groot K Macroporous biphasic calcium phosphate scaffold with high permeability/porosity ratio Tissue Eng 2003 9 535 548 12857421 10.1089/107632703322066714

[B31] Pennella F Cerino G Massai D Gallo D Falvo D’Urso Labate G Schiavi A Deriu MA Audenino A Morbiducci U A survey of methods for the evaluation of tissue engineering scaffold permeability Ann Biomed Eng 2013 41 2027 2041 23612914 10.1007/s10439-013-0815-5

[B32] Lee KW Wang S Lu L Jabbari E Currier BL Yaszemski MJ Fabrication and characterization of poly (propylene fumarate) scaffolds with controlled pore structures using 3-dimensional printing and injection molding Tissue Eng 2006 12 2801 2811 17518649 10.1089/ten.2006.12.2801

[B33] Al-Munajjed AA Hien M Kujat R Gleeson JP Hammer J Influence of pore size on tensile strength, permeability and porosity of hyaluronan-collagen scaffolds J Mater Sci Mater Med 2008 19 2859 2864 18347950 10.1007/s10856-008-3422-5

[B34] Mansour JM Mow VC The permeability of articular cartilage under compressive strain and at high pressures J Bone Joint Surg Am 1976 58 509 516 1270471

[B35] Lai WM Mow VC Drag-induced compression of articular cartilage during a permeation experiment Biorheology 1980 17 111 123 7407341 10.3233/bir-1980-171-213

[B36] O’Brein FJ The effect of pore size on permeability and cell attachment in collagen scaffolds for tissue engineering Technol Health Care 2007 15 15 17264409

[B37] Cohen NP Foster RJ Mow VC Composition and dynamics of articular cartilage: structure, function, and maintaining healthy state J Orthop Sports Phys Ther 1998 28 203 215 9785256 10.2519/jospt.1998.28.4.203

[B38] Allan KS Pilliar RM Wang J Grynpas MD Kandel RA Formation of biphasic constructs containing cartilage with a calcified zone interface Tissue Eng 2007 13 1 167 177 17518590 10.1089/ten.2006.0081

[B39] Khanarian NT Jiang J Wan LQ Mow VC Lu HH A hydrogel-mineral composite scaffold for osteochondral interface tissue engineering Tissue Eng A 2012 18 533 545 10.1089/ten.tea.2011.0279 PMC3286822 21919797

[B40] Moutos FT Guilak F Composite scaffolds for cartilage tissue engineering Biorheology 2008 45 501 512 18836249 PMC2727640

[B41] Yoshikawa H Myoui A Bone tissue engineering with porous hydroxyapatite ceramics J Artif Organs 2005 8 131 136 16235028 10.1007/s10047-005-0292-1

[B42] LeGeros RZ Properties of osteoconductive biomaterials: calcium phosphates Clin Orthop Relat Res 2002 395 81 98 11937868 10.1097/00003086-200202000-00009

[B43] Spivak JM Hasharoni A Use of hydroxyapatite in spine surgery Eur Spine J 2001 10 Suppl 2 S197 S204 11716019 10.1007/s005860100286 PMC3611554

[B44] Rezwan K Chen QZ Blaker JJ Boccaccini AR Biodegradable and bioactive porous polymer/inorganic composite scaffolds for bone tissue engineering Biomaterials 2006 27 3413 3431 16504284 10.1016/j.biomaterials.2006.01.039

[B45] Hench LL Polak JM Third-generation biomedical materials Science 2002 295 1014 1017 11834817 10.1126/science.1067404

[B46] Yang F Murugan R Wang S Ramakrishna S Electrospinning of nano/micro scale poly (L-lactic acid) aligned fibers and their potential in neural tissue engineering Biomaterials 2005 26 2603 2610 15585263 10.1016/j.biomaterials.2004.06.051

